# The Posterodorsal Medial Amygdala Regulates the Timing of Puberty Onset in Female Rats

**DOI:** 10.1210/en.2015-1366

**Published:** 2015-08-07

**Authors:** X. F. Li, M. H. Hu, B. P. Hanley, Y. S. Lin, L. Poston, S. L. Lightman, K. T. O'Byrne

**Affiliations:** Division of Women's Health (X.F.L., M.H.L., B.P.H., Y.S.L., L.P., K.T.O.), Faculty of Life Sciences and Medicine, King's College London, Guy's Campus, London SE1 1UL, United Kingdom; and Henry Wellcome Laboratory for Integrative Neuroscience and Endocrinology (S.L.L.), University of Bristol, Bristol BS1 3NY, United Kingdom

## Abstract

Obesity is the major risk factor for early puberty, but emerging evidence indicates other factors including psychosocial stress. One key brain region notable for its role in controlling calorie intake, stress, and behavior is the amygdala. Early studies involving amygdala lesions that included the medial nucleus advanced puberty in rats. More recently it was shown that a critical site for lesion-induced hyperphagia and obesity is the posterodorsal subnucleus of the medial amygdala (MePD), which may explain the advancement of puberty. Glutamatergic activity also increases in the MePD during puberty without a corresponding γ-aminobutyric acid (GABA)ergic change, suggesting an overall activation of this brain region. In the present study, we report that neurotoxic lesioning of the MePD advances puberty and increases weight gain in female rats fed a normal diet. However, MePD lesioned rats fed a 25% nonnutritive bulk diet also showed the dramatic advancement of puberty but without the increase in body weight. In both dietary groups, MePD lesions resulted in an increase in socialization and a decrease in play fighting behavior. Chronic GABA_A_ receptor antagonism in the MePD from postnatal day 21 for 14 days also advanced puberty, increased socialization, and decreased play fighting without altering body weight, whereas glutamate receptor antagonism delayed puberty and decreased socialization without affecting play fighting. In conclusion, our results suggest the MePD regulates the timing of puberty via a novel mechanism independent of change in body weight and caloric intake. MePD glutamatergic systems advance the timing of puberty whereas local GABAergic activation results in a delay.

The onset of puberty is heralded by the maturation of the hypothalamic-pituitary-gonadal axis, which is determined by a multiplicity of interrelated processes, although its mechanism remains unknown. Psychosocial stress can either delay or advance puberty both in humans and experimental animals (1–4). The changes in the timing of menarche can result from both environmental and social contexts. Parental divorce or childhood sexual or physical abuse accelerates menarche (1, 4), whereas social context has been shown to result in earlier menarche in higher ranking monkeys (3) and later ovulation in lower ranking females (3, 5). We have also shown that mimicking stress by the overexpression of corticotropin releasing factor in the central nucleus of the amygdala results both in anxiety and advancement of puberty in female rats (2). Numerous brain regions have been implicated in these stress-related neural circuits, with a prominent role suggested for the amygdala (6). The amygdala is also strongly implicated in social behaviors, especially play behavior and playing fight (eg, pinning), which are the earliest forms of nonmother-directed social behaviors and associated with prepubertal rats (7).

Amygdala lesions affect both fear (8) and the timing of puberty in female monkeys (5), and such lesions have been shown to alter the timing of puberty in rats from studies that date back to the 1960s (9, 10). Neurotoxic lesioning of whole amygdala at 1 month of age advanced puberty in female rhesus monkeys (5), whereas lesions at 10–13 months have no effect on menarche but delayed first ovulation in some monkeys (11). There are also controversies relating to the effects of amygdala lesions on pubertal timing in rats (10, 12, 13). Results from the large and unpredictable electrolytic lesions used in the rat studies may confound our understanding of amygdaloid organization because they do not take into account its heterogeneous nuclear arrangement and their related functions. For example, γ-aminobutyric acid (GABA) is associated with distinct roles in different subnuclei: aggression and anxiety with central nucleus of the amygdala (14, 15), fear extinction with basolateral amygdala (16), and social behaviors with medial amygdala (MeA) (17). However, the effect of lesioning specific subnuclei within the amygdala on puberty has not yet been established, and little is known of the potential impact on the pubertal timing of GABAergic or glutamatergic signaling in individual amygdala subnuclei.

The MeA is strongly implicated in ovulation, estrous cyclicity, gonadotropin secretion, and sexual behavior (18, 19). Gonadotropins increase after MeA lesion (20), whereas electrical stimulation delayed puberty in rats (21), suggesting this nucleus exerts an inhibitory influence on reproduction. The MeA, in particular its posterodorsal subnucleus (MePD) receives olfactory information (22), predominantly sends out inhibitory GABAergic projections and is implicated in reproductive behavior (22, 23). GABA is strongly implicated in fear and anxiety, with GABA_A_ receptors playing a major role (24–26). Stressors also alter the expression of GABA (27) and GABA_A_ receptors in the amygdala (28). Additionally, glutamate signaling in the amygdala, in particular its N-methyl-D-aspartate (NMDA) receptor, is involved in anxiety-related behavior (29), and intra-MePD administration of NMDA disrupts ovarian cyclicity in the rat (30). Whether NMDA or GABA_A_ receptor signaling in the MePD is involved in puberty timing has not been investigated.

Decrease in GABAergic and increase in glutamatergic tone in the hypothalamus are critical for initiating GnRH release, essential for puberty (31, 32). What is unknown, however, is the mechanism that regulates this hypothalamic tone. The MePD sends direct efferents to the GnRH-rich medial preoptic area (mPOA) (33, 34), which controls gonadotropin release. Therefore, the MePD is ideally situated to process environmental and sexual cues and bring about appropriate alterations in the reproductive axis. Additionally, the MePD has the highest density of sex steroid receptors of the amygdala and undergoes dramatic change during puberty, including increased glutamatergic activity, without a corresponding GABAergic change, suggesting an activation of this area at the onset of puberty (35). A critical site for lesion-induced hyperphagia and obesity in rats is also the MePD (36), which may explain the altered pubertal timing by way of an earlier attainment of critical body mass (37). The MeA is also implicated in the psychological stress-induced suppression of LH secretion in rats (38), which is of interest because psychological stress alters pubertal timing (1, 4).

In this study we tested the hypothesis that the MePD is critical for the timing of puberty and investigated whether this was independent of its regulatory role on weight gain. We examined the effect of MePD lesions on estrous cyclicity after puberty. We also assessed both social and aggressive behavior as proxy measures of psychological stress in MePD lesioned animals. Finally, we determined the role of the MePD GABA_A_ receptor, and the glutamatergic NMDA receptor in mediating changes in pubertal timing and behavior.

## Materials and Methods

### Animals housing, diets, and puberty evaluation

Late pregnant Sprague Dawley rats were purchased from Charles River and were housed under controlled conditions (12 h light, 12 h dark cycle, lights on at 7:00 am; temperature 22 ºC ± 2ºC), and supplied with food and water ad libitum. Litters were reduced to 10–12 pups on postnatal day (pnd) 1 (birth, day 0). They were weaned on pnd 21 and housed in groups of three to four per cage and weighed every 3 days. To determine whether the advancement of puberty seen in the lesion group could be explained by a change in body weight gain as previously reported (36), litters were assigned to either standard chow diet (RM1; Special Diet Services) or a nonnutritive bulk diet (the same standard chow to which 25% alphacel nonnutritive bulk [MP Biomedicals] had been added) immediately after being weaned. They were also monitored daily for vaginal opening and first vaginal estrus (markers of puberty onset) from pnd 28. Once vaginal opening occurred, vaginal smears were taken to detect the stage of the estrous cycle. For the MePD lesion and sham lesion control animals, the vaginal smears were taken daily for 12 consecutive days to determine the normality of the estrous cycles. Normal estrous cyclicity was defined as having at least two consecutive normal cycles, which lasts for 4–5 days with 1–2 days of estrus. Cycle length, with three stages observed in the correct order, was determined by the number of days between each occurrence of estrus.

### Surgical procedures

All surgeries on the prepubertal rats were carried out under a combination of ketamine (Vetalar, 600 mg/kg, ip; Pfizer) and xylazine (Rompun, 60 mg/kg, ip; Bayer). All procedures were conducted in accordance with the United Kingdom Home Office Regulations.

#### Bilateral lesions of the MePD

Bilateral lesions of the MePD (n = 28) were accomplished by the administration 0.2 μL of 10 μg/μL ibotenic acid (Sigma-Aldrich Ltd) in sterile 0.1 M PBS (pH 7.4). Ibotenic acid, an excitatory neurotoxic amino acid, produces selective lesions of the neuron cell bodies without destruction of fibers of passage. On pnd 21, pups were placed in a Kopf stereotaxic frame, and holes were drilled bilaterally in the skull at a location above the MePD after a midline incision was made in the scalp. The coordinates, 2.5 mm posterior to bregma (AP), 3.2 mm lateral (ML), and 7.8 mm below the surface of the dura (DV), were determined after careful optimization in a group of same-age pups in a preliminary experiment and used to target the MePD (AP: −3.3 mm; ML: 3.4 mm; DV: 8.6 mm) according to the procedures outlined in the rat brain atlas of Paxinos and Watson (39). A glass pipette connected to a tip of a 1-μL Hamilton microsyringe preloaded with the ibotenic acid was lowered into the target site over a 1-minute period, and a further 1 minute elapsed before the injection was initiated. The ibotenic acid was slowly infused into the nucleus over a 5-minute period, after which the glass pipette was left in place for an additional 5 minutes to prevent reflux. After being raised up slowly over 1 minute, the procedure was then repeated to lesion the nucleus on the other side. The scalp was closed with suture. After being removed from the stereotaxic frame, the rats were allowed to recover on a heated pad until fully conscious. The sham lesions were carried out using the same procedure but with the animals receiving sterile 0.1 M PBS (n = 17).

### Osmotic minipump implantation with GABA and glutamate antagonist administration

On pnd 21, standard chow diet-fed female rats were chronically implanted with brain cannulae. Animals were fitted bilaterally with 28-gauge cannulae (Plastics One) directed towards the MePD: coordinates used for implantation were the same as for MePD lesion described above. An osmotic minipump (model 1002; Alza Corp) prefilled with antagonists or artificial cerebrospinal fluid (aCSF) was attached to the cannula with silicone tubing, and implanted sc in the interscapular space. A glutamate antagonist, D-2-amino-5-phosphonovalerate (D-AP5, NMDA antagonist; Tocris) (n = 10), a GABA_A_ antagonist, bicuculline (n = 12) (Sigma-Aldrich), or aCSF (n = 7–10 per group) were delivered via the osmotic minipump at 0.25 μL/h for 14 days. The concentrations of D-AP5 and bicuculline used were 15 nmol/μL and 2.26 pmol/μL, respectively.

### Brain collection and histology verification of lesion site and cannula tip position

Once vaginal estrus was detected after the vaginal opening in the osmotic minipump implanted animals, they were killed by decapitation. The MePD-lesioned and sham-lesioned animals were killed by decapitation 12 days after the vaginal opening. The brains were removed and snap frozen on dry ice. Brains were stored at −80ºC and coronally sectioned (30 μm) at a later date using a cryostat (Bright lnstrument Co Ltd). To evaluate the lesion sites, every fourth section throughout the MePD region corresponding to bregma −2.80 to −3.60 mm (39) was mounted and stained with cresyl violet. Slides were then viewed under a light microscope, and images were taken using a digital camera (Zeiss) attached to the microscope. The boundaries of the MePD and the cannulae placement were determined using neuroanatomical landmarks compared with the rat brain atlas (39). Animals with a lesion or cannulae outside the desired structure were excluded from subsequent analysis.

### Effects of MePD lesion or intra-MePD administration of GABA and glutamate receptor antagonist on anxiety behavior

Three days before the first social interaction or play fighting behavior test was initiated, the rotarod test was used to evaluate motor function (40). The social interaction (41) and play fighting behavior (42) tests were performed as described previously to assess anxiety.

### Social interaction

Before starting the social interaction test, animals were habituated twice (once per day, 2 d before the day of testing) to the behavior test room for 15 minutes and then to the social interaction arena for 5 minutes on each occasion. Nine days after the microinjection of the ibotenic acid into MePD or implantation of the cannulae for the osmotic minipump delivery of GABA or glutamate antagonist into the MePD, the social interaction test was carried out once only. The time spent on the behavioral events (sniffing, chasing, following, grooming, and mounting) were recorded over a period of 15 minutes by two observers blind to the treatment. The tests were carried out between 9:00 and 11:00 am with the pairs of unfamiliar rats, weight matched and from the same treatment, placed simultaneously in the 50- × 50- × 50-cm arena. The total social interaction time was calculated by the sum of the time spent in the active social behaviors noted above.

### Play fighting behavior

On the day of the experiment, animals were socially isolated in cages for 2–3 hours prior to testing (42). The test consisted of placing two animals into a novel cage in which they were observed under a dim red light for a 20-minute period between 10:00 and 11:00 am. This procedure was carried out on pnd 27, 29, 31, and 33 in the MePD lesion and sham-lesioned animals but on pnd 29 and 31 for the intra-MePD administered GABA or glutamate antagonist animals. Each pair of tested animals was from an identical treatment group (lesion against lesion, sham against sham) and on the same diet regimen (42). The behaviors recorded included the following: pouncing (one rat lunges at another with its forepaws extended outward: this is initiation of play fighting); wrestling (two rats roll and tumble with each other); boxing (two animals standing upright facing and making pawing attack to each other); and pinning (the most characteristic posture of play fighting behavior, in which one of the animals is lying with its back on the floor of the cage and the other is standing over it, pinning it down) (42). Play fighting was defined as a summation of the duration of the play fighting behaviors listed above and averaged for the four 20-minute observation periods. In addition, the frequency of pinning events in the four 20-minute observation periods was quantified.

### Statistical analysis

Comparisons between rats that received ibotenic acid and sham lesions, GABA, or glutamate antagonist administration and their vehicle controls in the MePD with respect to body weight, day of vaginal opening, and first estrus and/or behavioral parameters were made using the ANOVA followed by post hoc Tukey-Kramer test, where appropriate. A percentage of normal estrous cycles was compared using Fisher's exact test. Data are presented as mean ± SEM, and *P* < .05 was considered statistically significant.

## Results

### Ibotenate lesioning results in specific neuron loss in the MePD

**Figure 1.**
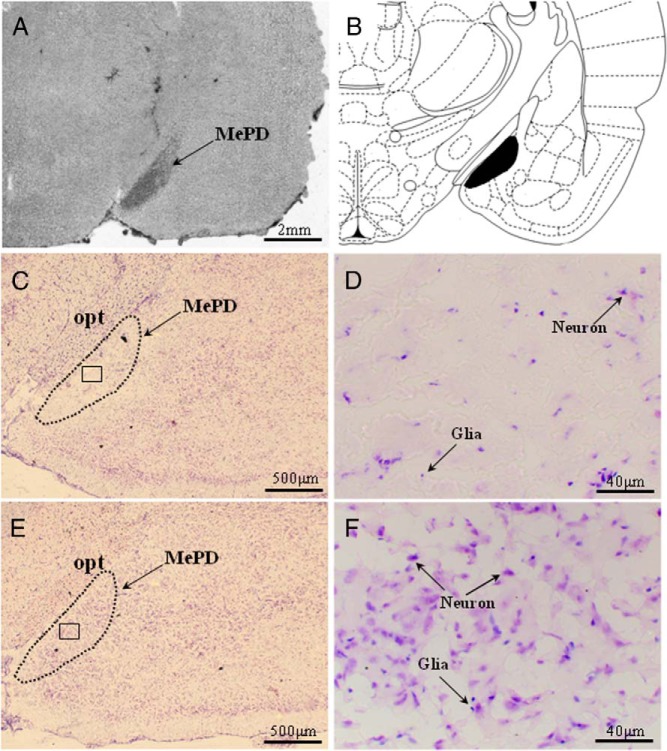
Coronal sections through the rat brain at the level of the MePD showing its spatial relationship with the surrounding nuclei and ibotenate lesion resulting in specific neuronal cell loss. A, Section without staining showing the lesion area localized to the MePD. B, Schematic representation of site of lesion in the MePD (black filled) adapted from the rat brain atlas (39). C, Representative example of a coronal brain section stained with cresyl violet illustrating substantial neuron loss in the MePD after ibotenic acid lesioning. The dotted line indicates the approximate border of MePD. E, Representative photomicrograph from a MePD sham-lesioned rat. D and F, Magnified images of the areas enclosed by the squares in panels C and E, respectively, and illustrating the significant neuron loss to the MePD (D). opt, optic track.

The extent and location of the lesions were confirmed by microscopic histological inspection, using cresyl-violet staining. The presence of extensive neuronal loss within the MePD was used as parameters to determine the existence of significant lesions. Only bilateral lesions that destroyed the MePD while leaving surrounding brain tissue largely intact were included in the study (Figure 1, A, C, and D). Animals were excluded from the analysis as a result of unsuccessful lesioning, unilateral damage, or damage extending outside the MePD. Of the 28 rats that underwent neurotoxic lesion surgery, 19 were verified with correct bilateral lesions in the MePD and were included in the analysis. The remaining nine were excluded. Of the 19 animals with correct lesions, eight were fed with the nonnutritive bulk diet and 11 with a standard diet. The sham lesion group contained seven fed with nonnutritive bulk food and 10 with a normal diet. All vehicle-injected rats sustained no obvious damage to the MePD (Figure 1, E and F).

### Effect of MePD lesions on body weight gain, timing of puberty onset, and estrous cyclicity

**Figure 2.**
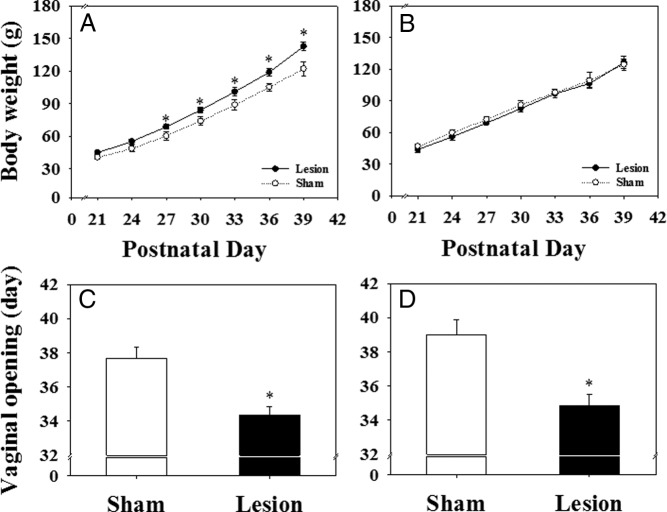
The effect of MePD lesions on body weight gain and day of vaginal opening in rats. A, Cumulative body weight gain was significantly greater from pnd 27 onward in the lesioned group compared with the sham-lesioned female rats fed with normal chow diet. B, In contrast, there is no significant difference in body weight gain between the lesioned and sham-lesioned animals fed the 25% nonnutritive bulk diet. The MePD lesion resulted in a significant advancement of pubertal onset (vaginal opening) in both normal (C) and nonnutritive bulk diet (D) fed rats. *, *P* < .05 vs sham lesion control. Results represent mean ± SEM (n = 7–11 per group).

Although there was no significant difference in body weight (Figure 2, A and B) at weaning among the experimental groups, MePD lesioning on pnd 21 increased body weight gain in animals fed normal standard diet, and this was evident from pnd 27 onward (Figure 2A; *P* < .05). Unsurprisingly, this was associated with a significant advancement of puberty onset compared with sham-lesioned controls (vaginal opening: 34.4 ± 0.5 vs 37.8 ± 0.7 d, respectively; Figure 2C; n = 10–11 per group; *P* < .05; first estrus: 34.6 ± 0.7 vs 37.9 ± 0.7 d, respectively, mean ± SEM; *P* < .0.05).

Feeding with a 25% nonnutritive bulk diet completely blocked the MePD lesion-induced increase in body weight (Figure 2B) but failed to prevent the advancement of puberty onset (vaginal opening: 34.9 ± 0.7 vs 39.0 ± 0.9 d, respectively; Figure 2D; n = 7–8 per group; *P* < .05; first estrus: 35.5 ± 0.7 vs 39.4 ± 0.8 d, respectively, mean ± SEM; *P* < .05).

**Figure 3.**
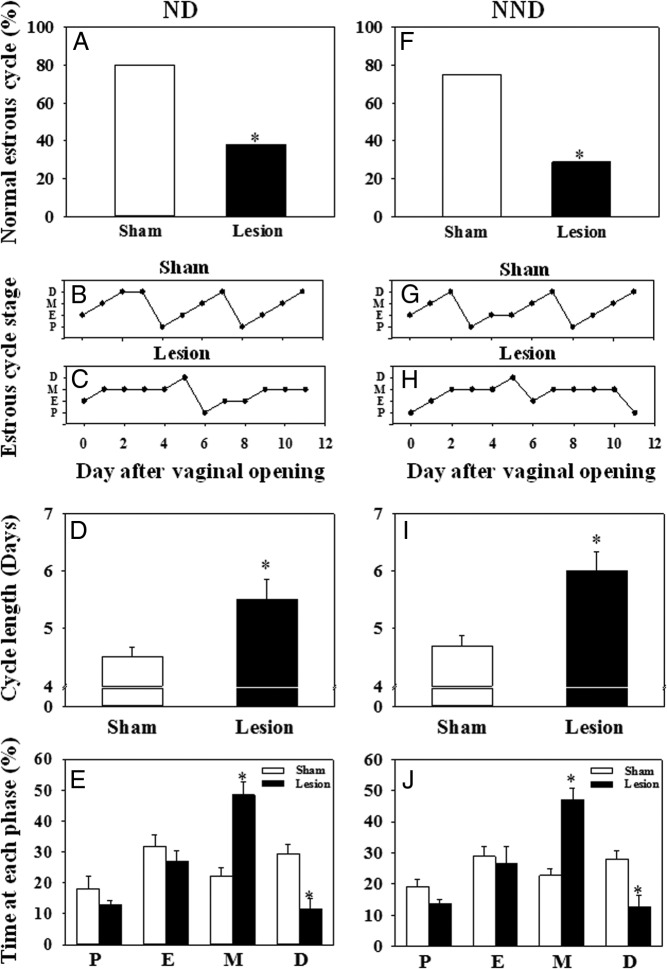
The effect of MePD lesions on estrous cyclicity in female rats fed with a normal chow diet (ND) or a 25% nonnutritive bulk diet (NND). Cyclicity was determined by analyzing the daily vaginal cytology for 12 consecutive days from the day of vaginal opening. A, In ND-fed rats, an MePD lesion resulted in a significant decrease in the percentage of normal estrous cycle vs sham-lesioned controls. Representative examples of estrous cycle are presented for sham-lesioned (B) and lesioned (C) rats. D, Cycle length was significantly extended after MePD lesion vs controls. E, Time spent in the metestrous stage was extended, whereas time spent in diestrus was decreased by MePD lesion compared with controls. F, Lesioning the MePD in NND fed rats also resulted in a significant decrease in the percentage of normal estrous cycle vs sham-lesion controls. Representative examples of the estrous cycle are presented for sham-lesioned (G) and lesioned (H) rats. I, MePD lesions also significantly extended cycle length compared with sham lesions in the NND fed rats. J, Time spent in the metestrous stage was extended, whereas time spent in diestrus was decreased by the MePD lesion compared with controls. Results are presented as means ± SEM. *, *P* < .05 vs controls (means ± SEM; n = 7–11 per group). D, diestrus; E, estrus; M, metestrus; P, proestrus.

Estrous cyclicity was disrupted in the immediate postpubertal period of the MePD lesioned rats fed either normal diet or nonnutritive bulk diet (Figure 3; *P* < .05). Most the control rats showed normal 4- to 5-day estrous cycles (80% of the normal diet group, n = 10; 75% of the nonnutritive bulk diet group, n = 7), whereas only 38% for the normal diet group (n = 11) or 29% for the nonnutritive bulk diet group (n = 8) of the MePD-lesioned animals showed normal estrous cycles (Figure 3, A and F; *P* < .05). Representative examples of estrous cycles from each group are illustrated in Figure 3, B, C, G, and H. Cycle length was prolonged (Figure 3, D and I; *P* < .05) with an increase in metestrus and a decrease in diestrous phases evident in MePD-lesioned rats fed with normal diet or nonnutritive bulk diet (Figure 3, E and J; *P* < .05).

### Behavioral effect of MePD lesion

Rotarod analysis revealed no difference between MePD-lesioned animals and their sham-lesioned controls in the time the rats stayed on the rotating rod (data not shown), indicating an absence of impaired motor function.

#### Social interaction

**Figure 4.**
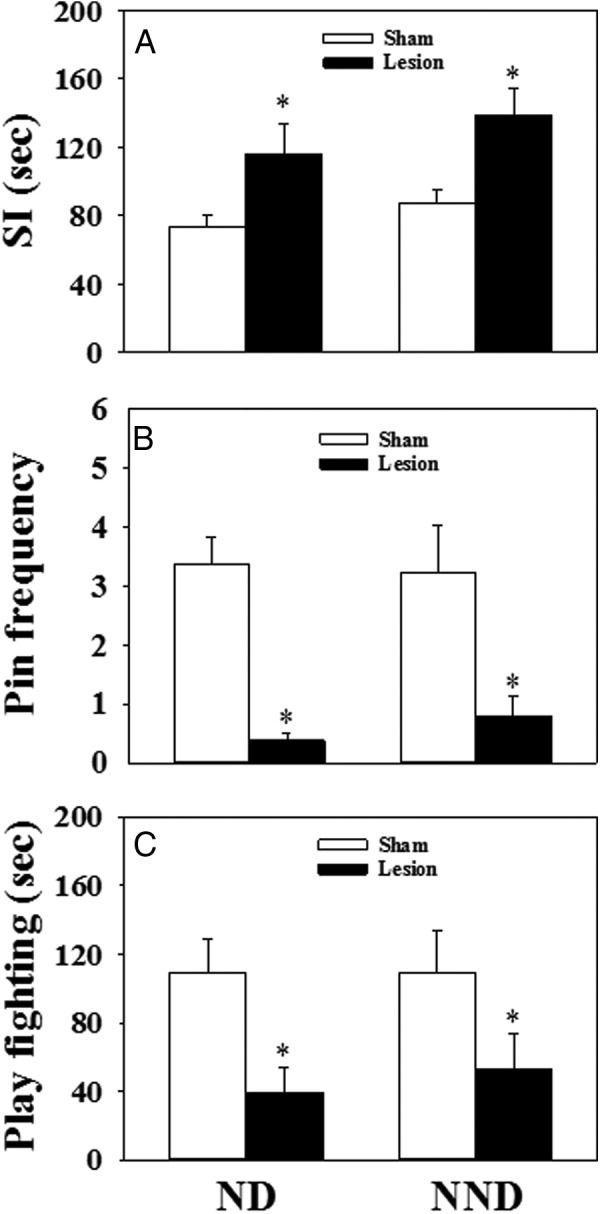
The effect of MePD lesions on the behavioral activities monitored between pnd 27 and 33 in female rats fed with normal chow diet (ND) or 25% nonnutritive bulk diet (NND). A, In the 15-minute social interaction (SI) test, the MePD lesion significantly increased the time of behavioral interaction (sniffing, chasing, following, grooming, and mounting) in both the ND and NND groups. For the 20-minute play fighting behavioral test, the lesion significantly decreased the frequency of pinning events per 20-minute observation period (Pin frequency) (B) and the duration of play fighting behavior (pouncing, wrestling, boxing, and pinning) (C) in both ND and NND fed rats. *, *P* < .05 vs sham lesion control. Results represent mean ± SEM, averaged for behavioral tests carried out on pnd 27, 29, 31, and 33 (n = 7–11 per group).

The social interaction testing was performed on pnd 30. There was a marked impact of lesioning the MePD on social behavior, with a significant increase in the cumulative mean time spent engaged in social interaction behavior, including sniffing, chasing, following, grooming, and mounting in the MePD-lesioned compared with the sham-lesioned controls, irrespective of diet (normal diet: 73.2 ± 6.9 vs 116.5 ± 17.6 sec, control vs lesion, n = 10–11 per group, *P* < .05; nonnutritive bulk diet: 87.4 ± 8.3 vs 138.3 ± 1.7 sec, control vs lesion, n = 7–8 per group, mean ± SEM, *P* < .05; Figure 4A).

#### Play fighting behavior

The frequency of pinning events was significantly decreased in the lesion animals in both the normal- and nonnutritive bulk diet-fed groups compared with their respective sham-lesioned controls (*P* < .01). The average number of pinning events recorded during the observation periods between pnd 27 and 33 in the sham lesion groups fed a normal diet and a nonnutritive bulk diet was 3.4 ± 0.5 and 3.2 ± 0.8 per 20 minutes, respectively (mean ± SEM) (Figure 4B). This decreased dramatically in the lesion animals fed a normal diet and a nonnutritive bulk diet to 0.4 ± 0.1 and 0.8 ± 0.3 per 20 minutes, respectively (Figure 4B). In the lesioned animals, the duration of play fighting behavior was remarkably decreased compared with sham-lesioned rats in both dietary food groups (normal diet: 109.6 ± 19.4 vs 39.3 ± 14.5 sec, control vs lesion, n = 10–11 per group, *P* < .05; nonnutritive bulk diet: 109.1 ± 24.3 vs 53.4 ± 19.9 sec, control vs lesion, n = 7–8 per group, mean ± SEM, *P* < .05; Figure 4C).

### Effect of glutamate or GABA antagonism in the MePD on puberty onset

**Figure 5.**
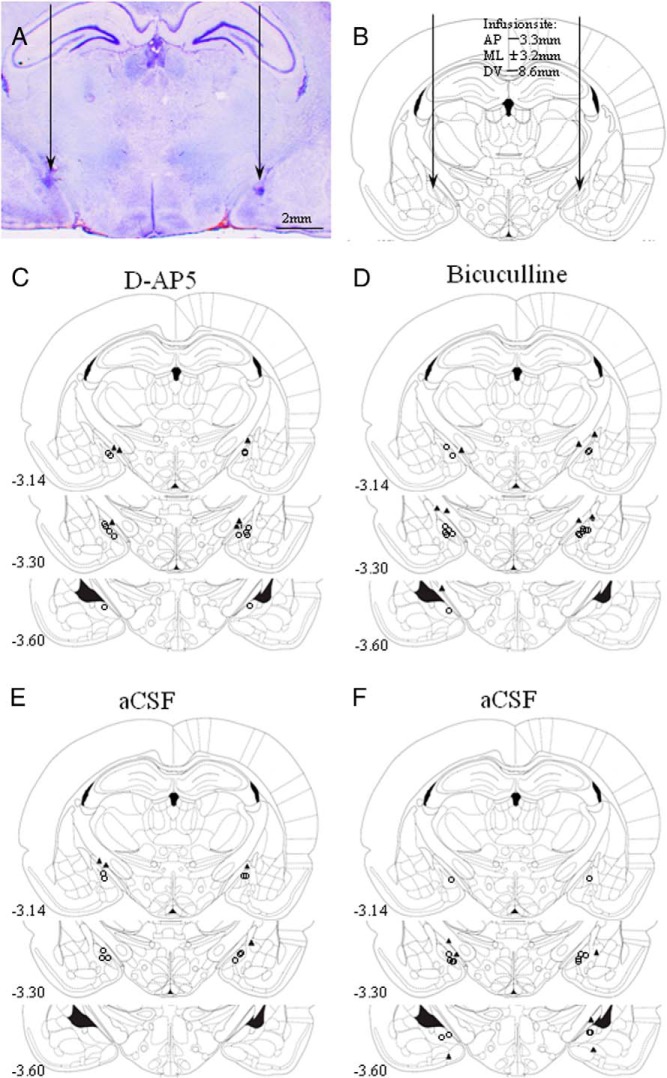
Photomicrograph and schematic illustrations of the osmotic minipump microinfusion sites targeted to the MePD. A, Photomicrograph of a coronal brain section in a representative animal implanted with bilateral cannulae in the MePD. Arrows indicate the site corresponding to the tip of the cannulae. B, Schematic illustration showing the target site for bilateral cannulation of the MePD at bregma (AP) of −3.3 mm, ML of ±3.4 mm, and DV of −8.6 mm according to the rat brain atlas of Paxinos and Watson (39). Arrows point to the location of the cannulae tips. C and D, Schematic drawings of the MePD illustrating the individual sites of chronic infusion of the D-AP5 or bicuculline, respectively. E and F, Schematic drawing of the MePD illustrating the individual sites of chronic infusion of aCSF as a control for the D-AP5 or bicuculline group, respectively. Open circles show the injection sites. Numbers in each drawing indicate the distance (millimeters) to bregma in accordance with Paxinos and Watson (39).

**Figure 6.**
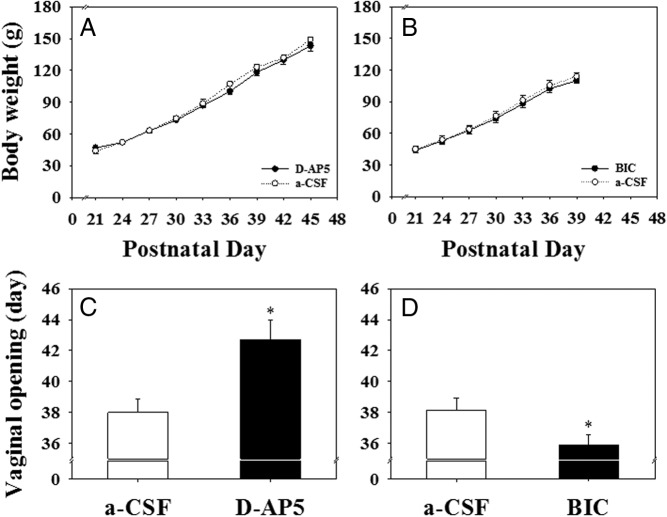
The effect of bilateral chronic microinfusion of D-AP5, BIC, or a-CSF (6 μL/d) in the MePD for 14 days starting on pnd 21 on body weight gain and the day of the vaginal opening in rats. A and B, There were no significant differences in cumulative body weight gain between the D-AP5 (90 nmol per 6 μL/d, bilaterally, for 14 d) or BIC (13.56 pmol per 6 μL/d, bilaterally, for 14 d) treatment groups and their corresponding aCSF controls. C, Chronic infusion of D-AP5 resulted in a significant delay of puberty onset. D, Chronic infusion of BIC in the MePD resulted in a significant advancement of puberty onset in female rats. *, *P* < .05 vs aCSF control. Results represent mean ± SEM (n = 5–8 per group).

Correct intra-MePD placement of cannulae connected to the osmotic minipumps filled with GABA or glutamate antagonists was verified with reference to the atlas of Paxinos and Watson (39), and histological analysis is displayed in Figure 5. A representative example is shown in Figure 5A. Schematic diagrams of coronal sections of the rat brain identifying the location of the cannulae tips for all rats that received intra-MePD infusions of D-AP5 (Figure 5C), bicuculline (Figure 5D), or their respective aCSF control (Figure 5, E and F) are illustrated. Chronic infusion of a glutamate antagonist, D-AP5 (90 nmol per 6 μL/d, bilaterally), into the MePD started on pnd 21 for 14 days and did not affect body weight gain (Figure 6A) but resulted in a significant delay of puberty (Figure 6C). The mean age of vaginal opening in the D-AP5 treatment group was significantly later than those controls infused with aCSF (42.7 ± 1.3 vs 38.0 ± 0.9 d, respectively, mean ± SEM; n = 5–7 per group; *P* < .05). However, bilateral chronic infusion of the GABA_A_ antagonist, bicuculline (13.56 pmol per 6 μL/d, bilaterally), into the MePD from pnd 21 to 35 caused a significant advancement of puberty (Figure 6D) without a change in body weight gain (Figure 6B). The mean age of vaginal opening in the bicuculline treatment group was significantly earlier compared with those animals treated with aCSF (35.9 ± 0.7 vs 38.1 ± 0.8 d, respectively, mean ± SEM; n = 7–8 per group; *P* < .05; Figure 6D).

### Behavioral effect of intra-MePD administration of GABA and glutamate antagonists

#### Social interaction

**Figure 7.**
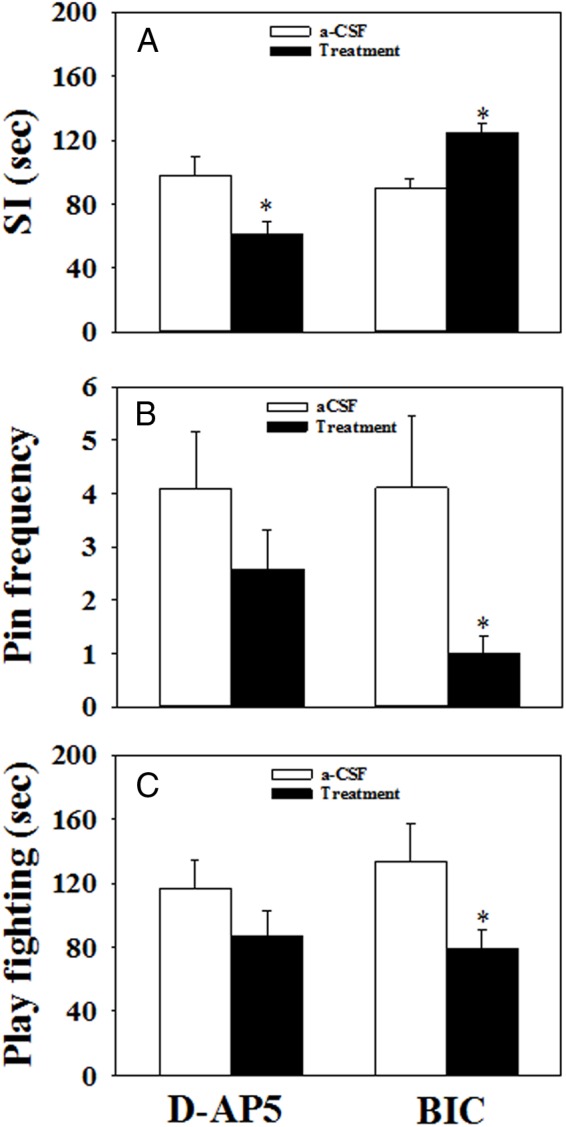
The effect of bilateral chronic microinfusion of D-AP5, bicuculline (BIC), or aCSF in the MePD on behavioral activities monitored between pnd 29 and 31 in female rats. A, In the 15-minute social interaction (SI) test carried out on pnd 30, chronic infusion of D-AP5 (90 nmol per 6 μL/d, bilaterally, for 14 d, starting on pnd 21) significantly decreased the time of behavioral interaction (sniffing, chasing, following, grooming, and mounting), whereas chronic infusion of BIC (13.56 pmol per 6 μL/d, bilaterally, for 14 d, starting on pnd 21) increased the interaction time significantly compared with controls (aCSF: 6 μL/d, bilaterally, for 14 d, starting on pnd 21). B, For the 20-minute play fighting behavioral test, there was no significant difference in the frequency of pinning events per 20-minute observation period (Pin frequency) between the D-AP5 and aCSF control-treated rats, whereas chronic infusion of BIC into the MePD results in a significant reduction of pin frequency. C, There was no significant difference in the duration of play fighting behavior (pouncing, wrestling, boxing, and pinning observed over a 20 min period) in D-AP5-treated rats, whereas BIC administration significantly decreased the duration of play fighting behavior compared with controls. *, *P* < .05 vs aCSF control. Results represent mean ± SEM, averaged for play fighting behavioral test carried out on pnd 29 and 31 (n = 5–8 per group).

The social interaction testing was performed on pnd 30. There was a marked impact of intra-MePD administration of D-AP5, with a significant decrease in the cumulative mean time (61.2 ± 7.8 sec, n = 7) spent engaged in social interaction behavior compared with the control (97.7 ± 11.6 sec, n = 5, mean ± SEM, *P* < .05; Figure 7A). On the contrary, chronic infusion of bicuculline into the MePD significantly increased the mean time spent engaged in social interaction behavior (bicuculline treatment vs control, 124.8 ± 5.5 vs 89.5 ± 6.3 sec, n = 7–8 per group, mean ± SEM, *P* < .05; Figure 7A).

#### Play fighting behavior

There was no significant effect of intra-MePD administration of D-AP5 administration on play fighting behavior (Figure 7, B and C). However, the frequency of pinning events was significantly decreased by bicuculline (*P* < .05). The average number of pinning events recorded during the observation periods on pnd 29 and 31 in the control and bicuculline treatment groups was 4.1 ± 1.1 and 1.0 ± 0.3 per 20 minutes, respectively (mean ± SEM) (Figure 7B). In the bicuculline-treated animals, the duration of the play fighting behavior was also decreased compared with the controls (133.8 ± 23.1 vs 79.2 ± 12.0 sec, aCSF vs bicuculline, n = 7–8 per group, mean ± SEM, *P* < .05; Figure 7C).

## Discussion

We have shown for the first time that a neurotoxic lesion specific to the MePD markedly advanced the timing of puberty onset in female rats and resulted in an average 17% weight gain by pnd 39. Previous studies in adult animals have also shown lesions of the MePD result in marked hyperphagia and excessive weight gain (36). The effect of MePD lesions on food intake and body weight is highly site specific because lesions more posteriorly through the amygdalohippocampal area or into the subiculum do not affect food intake or body weight (10). Damage to large areas of the amygdala, including the anterior, basolateral, and corticomedial group of nuclei, are also without effect (43). Similarly, in the present study, rats fed a standard diet with lesions to the anterior or ventral MeA did not show differences in body weight gain compared with sham controls (data not shown). Because it is thought that body weight influences the timing of puberty and a critical body weight is necessary for its onset (37), it is reasonable to assume the increase in body weight in MePD-lesioned rats may underlie the advancement of puberty in the present study. However, by feeding the MePD-lesioned animals a 25% nonnutritive bulk diet, we could tease apart whether the advancement of pubertal timing was related to this increased calorie intake and increased body weight gain. On the contrary, the lesioned rats fed the nonnutritive bulk diet had an identical dramatic advancement of puberty without the increased body weight. Moreover, in both dietary groups, puberty occurred in lesioned animals at a significantly lower body weight compared with controls because of their younger age, which is inconsistent with the critical body mass hypothesis (37). Therefore, our results suggest the MePD plays a critical and novel role in pubertal timing via mechanisms independent of change in body weight or caloric intake.

The amygdala is commonly known for its role in higher-order emotion processing that was initially suggested by Kluver and Bucy (44) in their classical temporal lobectomy studies in rhesus monkeys, which resulted in major changes in their emotional behavior, cumulatively termed psychic blindness. Essentially the monkeys became tame, fearless, and emotionally flattened. The MePD is considered part of an integrated neural circuit involved in responses to fear and stressful conditions and in modulating social and aggressive behaviors. As was expected, the MePD-lesioned rats showed a decrease in social play behavior during play fighting tests in the present study, which is consistent with previous data in rats which received amygdala lesions on pnd 7 or 21 (42). Activation in the MePD after restraint stress confirms its participation in psychological stress in rats (45). Exposure to predator (cat) similarly activates the rat MePD (46), indicating its functional relationship to anxiety or fear. The MePD is also robustly activated by social defeat (47) and exposure to aggressive social encounters in the juvenile social subjugation paradigm, which is considered an animal model for child abuse (48). In the present study, the marked impact of intra-MePD administration of D-AP5 decreasing social interaction behavior and the significant increase in social play and social interaction behavior in the rats treated with bicuculline suggest that the GABAergic and glutamatergic neuronal systems in the MePD may be key players in these behaviors. The MePD connects to specific hypothalamic nuclei, participates in interpretation of olfactory (49) and genitosensorial (50) signaling, and modulates social and sexual behavior in male and female rats (45, 51). The MePD sends direct efferents to the GnRH-rich mPOA (33, 34), and stimulation of the former induces GnRH release (18).

The MePD also projects to the hypothalamic anteroventral periventricular and arcuate nuclei, which contain key kisspeptin neuronal populations essential for gonadotrophic hormone secretion and puberty (52–54). In addition, it was recently discovered that extrahypothalamic kisspeptin neurons in the MePD are developmentally up-regulated between the juvenile and adult state (55), and we have recently shown that administration of a kisspeptin antagonist directly into the MePD profoundly suppresses LH pulse frequency in the female rats (56). Thus, in addition to hypothalamic kisspeptin, the MePD population may be involved in the timing of puberty.

The MePD also contains one of the highest concentrations of estrogen, progesterone, and androgen receptors (57). The MePD is therefore ideally positioned to integrate systems required to optimize behavioral adaptation and the timing of reproductive events in myriad environment. In fact, social rank is closely correlated to the age of puberty onset in nonhuman primates, with higher social rank related to earlier testicular development in males (58) and sexual skin swelling and menarche in females (58, 59). Neonatal amygdala lesions advance pubertal timing, although they delay first ovulation and concomitantly reduce fear and increase exploratory behavior in female rhesus monkeys (5, 8, 11). There is also evidence that psychosocial stress can delay or advance puberty in animals, including humans (1–4). These data support a key role for the MePD in stress-induced change in pubertal timing and support the contention that adaptive reproductive behavior necessitates integrating external signals about the suitability of the surrounding environment for reproduction with the internal control of the reproductive state (60). This is of interest here because the rats in the present study appear to be afflicted by a similar psychic blindness, evidenced by their increased playful socialization and loss of play fighting behavior. The advancement of puberty in this case might be that MePD lesion prevents the rat experiencing fear or prevents translation of the fear experience into a suppression of the reproductive axis. This might suggest that under normal circumstances, the MePD functions to inhibit puberty until such a time as the environment appears safe to allow for reproduction. However, such a causal relationship between these two aspects of MePD function awaits further investigation.

The mechanism whereby MePD lesions result in an early onset of puberty is unknown. However, we have shown in preliminary studies that the dramatic advancement of puberty in the MePD-lesioned animals was associated with an earlier onset of higher LH pulse frequency (X.F.L., M.H.H., unpublished observation), suggesting an earlier acceleration of GnRH pulse generator frequency in the prepubertal period. It is well established that a decrease in GABAergic tone and increase in glutamatergic tone in the hypothalamus are critical for pubertal timing in rodents and primates (31, 32, 61, 62). Prior to puberty, there is a decline in GABA release concomitant with increased GnRH release (63). Chronic microinfusion of the GABA_A_ receptor antagonist, bicuculline, into the hypothalamus significantly increased GnRH release and accelerated the timing of puberty in female monkeys (64). More recently it was demonstrated that GABA_A_ receptor antagonism dramatically stimulated kisspeptin release in the mediobasal hypothalamus of prepubertal monkeys and that bicuculline-induced GnRH release is blocked by kisspeptin receptor antagonism, which suggest that kisspeptin neurons may relay inhibitory GABAergic signals to GnRH neurons (31). Similarly, glutamatergic signaling plays an important role in pubertal timing. NMDA induces GnRH release, resulting in precocious puberty in rats (62) and monkeys (61), whereas NMDA receptor antagonism delays puberty (65). However, what regulates hypothalamic GABAergic or glutamatergic tone remains to be established. The MePD undergoes dramatic change during puberty in rodents, including volumetric growth, due in part to increased neuronal soma size and dendritic spine density and increased glutamatergic synaptic activity but without an apparent corresponding GABAergic change, which indicates an overall increased excitability and activation of this area at the onset of puberty (35, 66, 67).

The volume of gray matter within the human amygdala also increases during puberty, and these morphometrics are associated with changing levels of circulating sex steroids (68). The delay of puberty after antagonism of the NMDA receptors in the MePD supports a role for amygdala glutamatergic signaling in stimulating puberty onset. However, these data are seemingly inconsistent with the advancement of puberty after discrete lesions of the MePD. One explanation for this conundrum would be the lesion causes loss of inhibitory GABAergic outputs, which constitute the majority (50%–70%) of MePD projection neurons (69). This may result in release of a tonic inhibitory break exerted by these amygdala projections on key hypothalamic reproductive nuclei, including the GnRH-rich mPOA (33, 34), and kisspeptin-rich anteroventral periventricular and arcuate nuclei (22, 23, 52), although the neuronal phenotypes of these direct projection fibers and whether they contact these essential reproductive neurons remains to be established. We have, however, previously shown the MeA suppresses pulsatile LH secretion through activation of GABAergic neurons intrinsic to the mPOA (38), adding to the complexity of amygdaloid control of gonadotropic hormone secretion. Because the glutamate component of the MePD is considerably smaller than GABA, which constitutes 70% of the total nuclear cell count (22, 69), this might suggest a differential dominance of inhibition in this nucleus. Our data suggest the balance between GABA and glutamate function intrinsic to the MePD is crucial in triggering puberty onset because their antagonism advances and delays puberty, respectively. Nevertheless, the hierarchical and developmental relationship between MePD GABA and glutamate systems, both the intrinsic and extrinsic projecting components, in modulating pubertal timing remains to be established.

In the present study, lesioning the MePD disrupted estrous cyclicity including a lengthening of the cycle in the immediate postpuberty period. The time spent in metestrus was profoundly increased in addition to a reduction in diestrus in MePD-lesioned rats. This may indicated abnormal levels of estrogen or progesterone caused by abnormal follicle development (70). Although it is reported that bilateral electrolytic lesioning of the MePD in adult rats did not affect estrous cyclicity, only the time taken to display estrus after surgery was recorded without an evaluation of cyclicity per se (71). However, a whole-amygdala lesion causes increased cycle length (72). The reason for the prolonged estrous cycles in the prepubertal MePD lesioned rats may be due to the disruption of ovulation because stimulation of the amygdala causes advancement of ovulation or resumption of estrous cycles after periods of prolonged diestrus (73). Additionally, lesion of the corticomedial amygdala blocks ovulation (74). Further studies will be necessary to establish whether prepubertal MePD lesions affect the preovulatory LH surge and whether the disruption to estrous cyclicity persists long term.

In conclusion, our results suggest that the MePD alters the timing of puberty onset via a mechanism independent of change in body weight and food intake. MePD glutamatergic systems advance the timing of puberty, whereas local GABAergic activation results in a delay.
